# Serum calcium dynamics and risk of all-cause hospitalization and mortality in hemodialysis patients: a landmark analysis from the international DOPPS

**DOI:** 10.1093/ckj/sfag324

**Published:** 2026-09-12

**Authors:** Ken Iseri, Taigi Yamazaki, Iori Taki, Noriko Hida

**Affiliations:** Department of Clinical Pharmacy, Division of Clinical Research and Development, School of Pharmacy, Showa Medical University, Tokyo, Japan; Jinsei-kai Kasai Dialysis Clinic, Tokyo, Japan; Department of Clinical Pharmacy, Division of Clinical Research and Development, School of Pharmacy, Showa Medical University, Tokyo, Japan; Department of Clinical Pharmacy, Division of Clinical Research and Development, School of Pharmacy, Showa Medical University, Tokyo, Japan; Department of Clinical Pharmacy, Division of Clinical Research and Development, School of Pharmacy, Showa Medical University, Tokyo, Japan

**Keywords:** calcium, hemodialysis, hospitalization, mortality, variability

## Abstract

**Background:**

Calcium is measured repeatedly in hemodialysis care, yet most studies characterize exposure using a single value rather than its temporal behavior. We examined whether recent serum calcium dynamics are associated with short-term hospitalization and mortality in maintenance hemodialysis patients.

**Methods:**

Using the Dialysis Outcomes and Practice Patterns Study phases IV–VII, we conducted a quarterly dynamic landmark analysis. At each landmark, total calcium values during the preceding 180 days were summarized as the latest value, mean, linear slope, residual standard deviation (rSD), and time-below-range (TBR, <8.6 mg/dl) and time-above-range (TAR, >10.0 mg/dl). All-cause hospitalization and death were assessed over the subsequent 90 days using discrete-time survival models.

**Results:**

Among 81 475 patients contributing 431 618 landmarks, 68 632 hospitalizations and 12 886 deaths occurred. Latest calcium was not associated with hospitalization [hazard ratio (HR), 1.00] but was associated with higher mortality (HR, 1.16). Lower mean calcium was associated with hospitalization (HR, 0.97), whereas higher mean calcium was associated with death (HR, 1.16). A positive slope and greater rSD were consistently associated with both hospitalization (HRs, 1.10 and 1.15) and death (HRs, 1.38 and 1.37). TBR burden showed graded associations with hospitalization, whereas TAR burden was strongly and dose-dependently associated with mortality.

**Conclusions:**

Recent calcium dynamics were associated with short-term outcomes in an outcome-specific manner: low calcium and TBR with hospitalization, high calcium and TAR with death, and rising or unstable calcium with both. These findings support longitudinal assessment of serum calcium, particularly its instability, which was not captured by a single value.

## Graphical Abstract

**Graphical Abstract:**
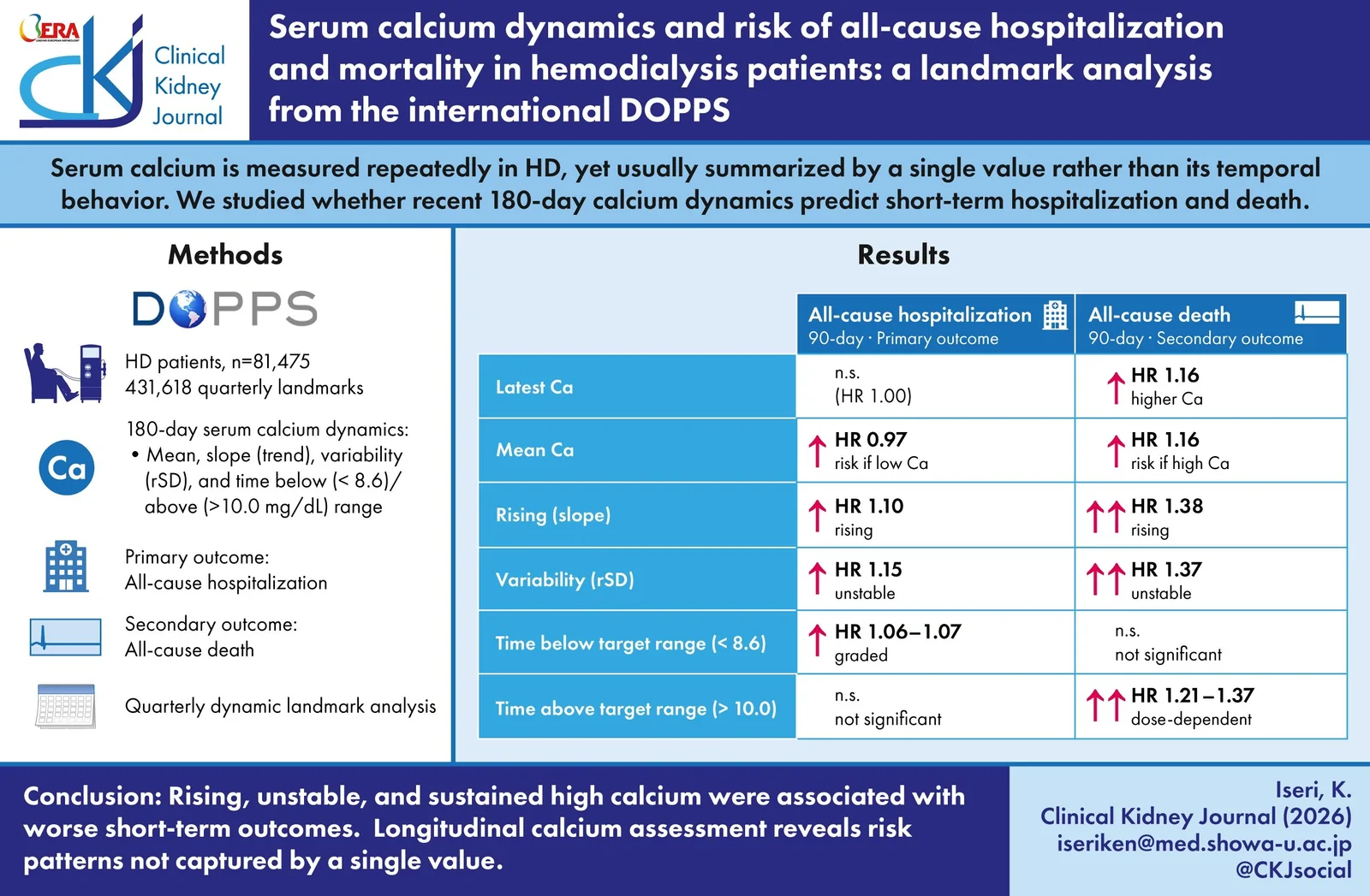

KEY LEARNING POINTS **What was known:**

- CKD-MBD disturbances are associated with morbidity and mortality in hemodialysis patients, and current guidance emphasizes serial assessment rather than single laboratory values.
- Previous Dialysis Outcomes and Practice Patterns Study (DOPPS) and registry studies suggested that serum calcium outside the usual target range, particularly hypercalcemia, is associated with adverse outcomes.
- Most prior studies characterized calcium exposure using baseline, latest, or time-averaged values, leaving the prognostic importance of recent calcium slope, variability, and time out of range uncertain

**This study adds:**

- In 81 475 hemodialysis patients contributing 431 618 landmarks from DOPPS data, recent 180-day calcium dynamics were associated with 90-day hospitalization and mortality.
- Lower mean calcium and greater time-below-range were mainly associated with hospitalization, whereas higher mean calcium and time-above-range were strongly associated with mortality.
- Rising calcium and greater calcium instability were consistently associated with both hospitalization and death, with findings robust to parathyroid hormone adjustment and corrected-calcium sensitivity analyses.

**Potential impact:**

- Routine hemodialysis care may benefit from reviewing calcium trends, variability, and time out of range, rather than responding only to the latest calcium value.
- Low or persistently below-range total calcium may identify patients who need assessment for illness, malnutrition-inflammation, frailty, or other causes of short-term hospitalization risk.
- Sustained high or rising calcium, marked variability, and prolonged time above range may warrant heightened surveillance as a signal warranting review of CKD-MBD management and clinical instability.

## INTRODUCTION

Chronic kidney disease-mineral and bone disorder (CKD-MBD) contributes substantially to morbidity and mortality in hemodialysis, and current guidance emphasizes therapeutic decisions based on serial assessments of calcium, phosphate, and intact parathyroid hormone (PTH) rather than a single laboratory value [1–5]. Large observational studies have consistently shown U- or J-shaped associations between CKD-MBD markers and mortality, and the management of CKD-MBD remains central to improving outcomes [6–11]. An earlier Dialysis Outcomes and Practice Patterns Study (DOPPS) analysis reported a total calcium of 8.6–10.0 mg/dl was associated with the lowest mortality, whereas values above 10.0 mg/dl showed the highest risk [8]. Comparisons of uncorrected with albumin-corrected calcium show that the apparent risk of low calcium is largely attenuated after albumin adjustment, whereas the risk associated with high calcium is strengthened [12], suggesting that low total calcium may partly reflect hypoalbuminemia or illness severity, whereas high calcium may more directly reflect mineral metabolism burden.

However, most prior studies have characterized calcium exposure using a single baseline value, the most recent value, or a broad time-averaged concentration, none of which captures the temporal behavior that clinicians actually observe and make treatment decisions. In routine hemodialysis care, serum calcium is measured repeatedly, and clinicians often respond not only to the single absolute value but also to whether calcium is persistently low or high, rising over time, or fluctuating markedly. Likewise, longitudinal data suggest that cumulative calcium exposure and changes in calcium over time provide prognostic information beyond any single measurement [13]. As for phosphate and PTH, dynamic features such as the mean, slope, and variability, together with time-in-range metrics, have already been linked to clinical outcomes [14–16]. By contrast, evidence for calcium remains sparse.

A single outcome may not fully capture how calcium relates to clinical risk because hospitalization and death may reflect different biological pathways. Low total calcium may be a marker of hypoalbuminemia, malnutrition-inflammation, frailty, or acute illness that predisposes to hospitalization. In contrast, sustained or rising high calcium may reflect calcium loading or low bone buffering capacity and may be more closely related to cardiovascular and fatal events. We therefore examined both endpoints to avoid overlooking outcome-specific calcium characteristics.

Thus, we used data from the international DOPPS to investigate whether recent 180-day calcium dynamics are associated with subsequent short-term all-cause hospitalization and all-cause death in maintenance hemodialysis patients. Using a quarterly dynamic landmark design, we characterized calcium exposure with complementary metrics: the latest total calcium value, the 180-day mean, the calcium slope, the residual standard deviation (rSD) as a measure of instability, and cumulative time-below-range (TBR) and time-above-range (TAR) burden. We further assessed whether these dynamic features carried prognostic information independent of the latest single calcium value, and whether the associations were robust to albumin correction and to adjustment for PTH.

## MATERIALS AND METHODS

### Data source

The DOPPS is an international prospective cohort of representative in-center hemodialysis patients [17]. Laboratory values and medication prescriptions are recorded monthly, less time-varying measures (e.g. single-pool Kt/V, body mass index) every 4 months, and comorbidities at baseline. Because the study used de-identified data, informed consent was not required. The study protocol using DOPPS data was approved by the Showa University Ethics Committee (2024-230-B).

### Participant selection and study design

This study used data from 131 269 hemodialysis patients enrolled in DOPPS Phases IV–VII (2009–22). Patients were excluded if they had no medication data, no serum total calcium measurement during observation, no laboratory or medication record on or after the DOPPS index date, or no follow-up time at risk, leaving 120 900 patients eligible for a quarterly dynamic landmark analysis (Fig. 1). For each patient, the first landmark was set 180 days after baseline, and subsequent landmarks were placed every 90 days thereafter. At each landmark, a 180-day look-back window was used to summarize calcium dynamics and time-varying covariates, and a 90-day forward window was used to ascertain outcome events; the forward window was divided into three consecutive 30-day intervals (days 1–30, 31–60, and 61–90) (Fig. 2). A patient re-entered the analysis at each subsequent landmark for which he or she remained at risk. A landmark was eligible for analysis if (i) it preceded the end of follow-up, so that at least one day of subsequent observation was available; (ii) the patient was not hospitalized on the landmark date; and (iii) serum total calcium had been measured in at least 4 months within the preceding 180 days, including at least one measurement within the preceding 45 days. After applying these criteria, 81 475 patients contributed 431 618 landmarks [median 5 landmarks per patient, interquartile range (IQR) 2–8].

**Figure 1:**
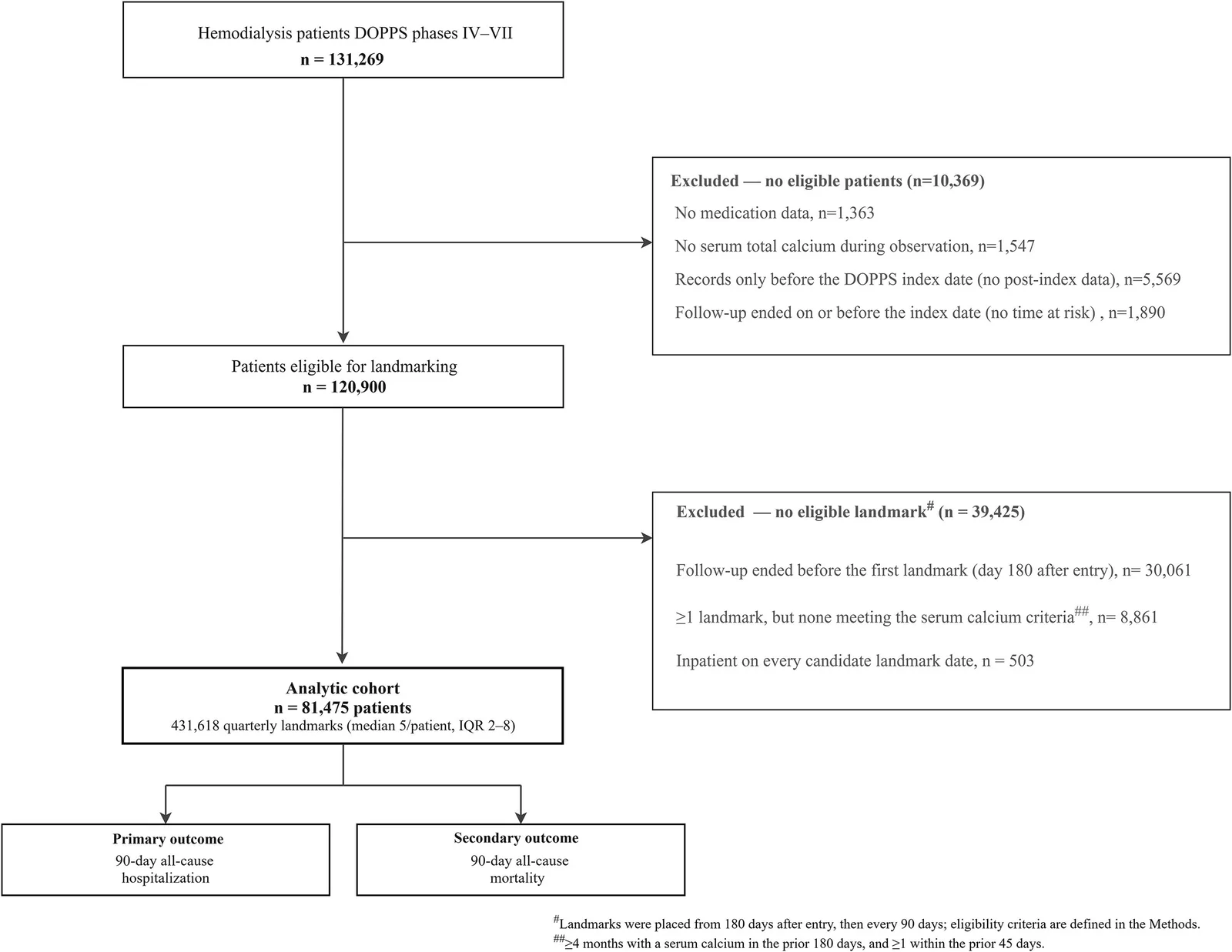
Patient flow chart.

**Figure 2:**
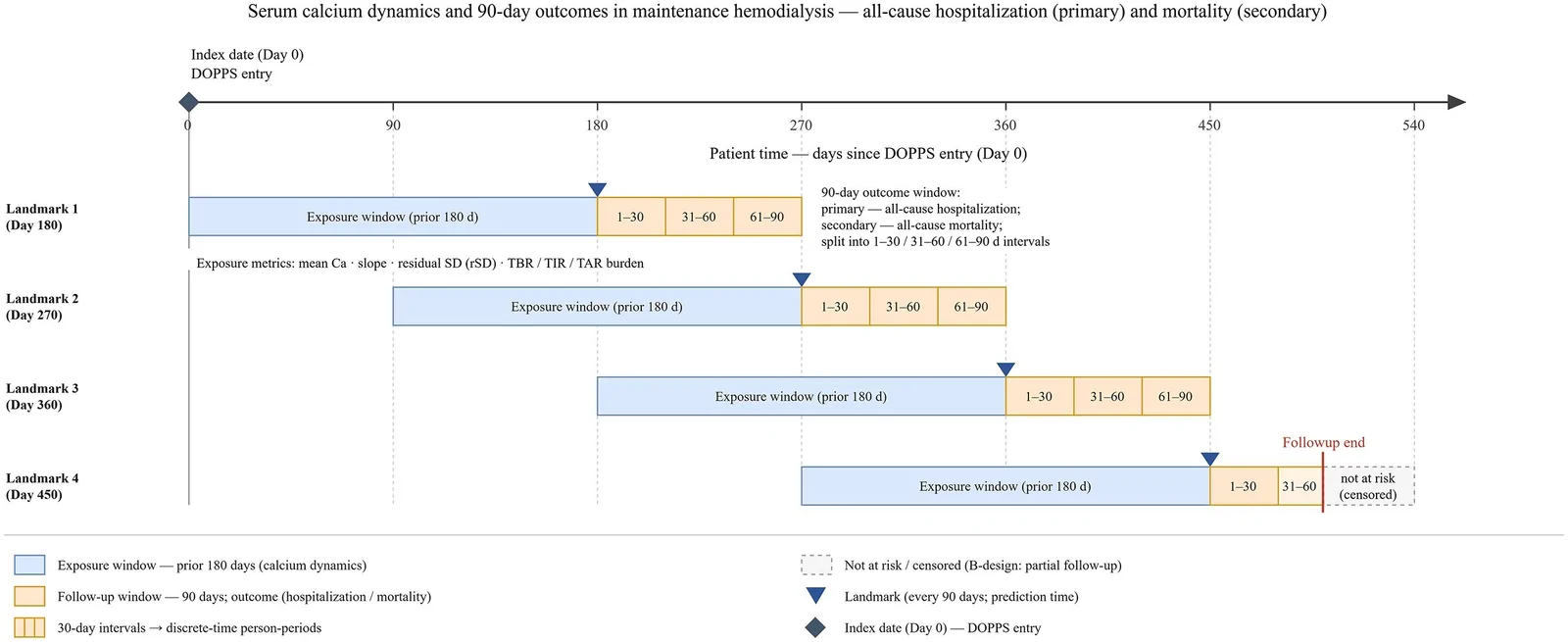
Quarterly dynamic landmark design.

### Definition of exposure

The primary exposure was the temporal dynamics of serum total calcium over the 180 days before each landmark, summarized as three jointly modeled features: the mean concentration (per +1 mg/dl), the slope of a least-squares regression of calcium on time (per +1 mg/dl per 30 days), and the residual SD about that regression line (rSD; per +1 mg/dl; computed when ≥3 values were available). Two prespecified alternative parameterizations were also examined: (1) time-in-range burden, classifying each monthly value as below (TBR, <8.6 mg/dl), within (TIR, 8.6–10.0 mg/dl), or above range (TAR, >10.0 mg/dl) and counting months in each zone (0, 1–2, or 3–6 months), with the 8.6–10.0 mg/dl range based on the lowest-mortality range in a prior DOPPS report [8]; and (2) Payne-corrected calcium [total Ca + 0.8 × (4.0 − albumin)], used to derive analogous mean, slope, and rSD features as a sensitivity exposure.

### Outcomes

The primary outcome was all-cause hospitalization and the secondary outcome was all-cause death, each within the 90-day window after a landmark. For each landmark, follow-up was censored at 90 days, at the outcome event, or at the end of the observation period, whichever occurred first. For the hospitalization outcome, follow-up additionally ended at the close of each patient’s hospitalization-data collection, whereas deaths were ascertained through the administrative end of follow-up.

### Covariates

All models adjusted for the same case-mix block: age, dialysis vintage, sex, race, country/region, DOPPS phase, and 12 baseline comorbidities. Time-updated laboratory covariates included albumin, phosphate, hemoglobin, creatinine, alkaline phosphatase, single-pool Kt/V, body mass index, and blood urea nitrogen. Time-updated treatment covariates included phosphate-binder category, dialysate calcium concentration, erythropoiesis-stimulating agents, vitamin D receptor activators, and calcimimetics. Prior hospitalization was captured by two prespecified terms in mutually exclusive windows: the number of admissions 31–180 days before the landmark and a binary indicator of any admission within the preceding 30 days (detailed in Item S1).

### Statistical analysis

Continuous variables are presented as mean ± SD or median (IQR), as appropriate, and categorical variables as *n* (%). The association between serum calcium dynamics and each outcome was estimated using a discrete-time survival model using a complementary log–log link applied to 30-day person-period data, including an interval-specific intercept, a facility-level random intercept, and an offset for partial follow-up; exp(β) was interpreted as the hazard ratio (HR) per 30-day interval (full specification in Item S1). Calcium metrics were entered as linear terms in the primary models and as natural cubic splines in prespecified secondary analyses (latest and mean calcium, 4 df; rSD, 3 df; reference, 9.3 mg/dl for concentrations and the median for rSD).

We used eight prespecified analytical models to assess the calcium–outcome associations through complementary exposure parameterizations. The models were constructed as follows:

✓ Model 1 (Minimally adjusted): Included only the latest total calcium and the case-mix block, omitting time-varying laboratory, medication, and prior-hospitalization covariates, and served as a reference for conventional cross-sectional associations.
✓ Model 1-full: Added the full time-varying covariate block to Model 1; the contrast between Model 1 and Model 1-full isolates confounding by nutritional and inflammatory markers, principally albumin.
✓ **Model 2** (**Primary model**): Replaced the single latest-calcium measurement with the three 180-day calcium-dynamics features (mean, slope, and rSD) and retained the full adjustment block.
✓ Model 3: Replaced the calcium-dynamics features with the categorical TBR and TAR burden variables.
✓ Model 4: Added the PTH (time-varying categorical variable) to Model 2.
✓ Model 5a (Primary sensitivity): Used the corrected-calcium dynamics (mean cCa, slope cCa, and rSD cCa); albumin was deliberately omitted from its adjustment block because corrected calcium is itself a function of albumin (Payne formula).
✓ Model 5b (Exploratory): Reintroduced albumin to Model 5a to evaluate the consequences of double-adjustment.
✓ Model 2_same: Refitted Model 2 on the restricted landmark set used in Model 4, in order to distinguish the sample-selection effect of PTH availability from the effect of adjusting for PTH itself.

All models shared the facility random intercept and the same case-mix block. Missing data were handled by complete-case analysis for each model (detailed in Item S1). Incremental predictive performance was assessed by comparing latest-calcium, calcium-dynamics, and augmented models using patient-grouped five-fold cross-validation, 90-day inverse-probability-of-censoring-weighted time-dependent area under the curve (AUC) and Brier score (Item S1). Furthermore, exploratory cause-specific analyses classified first hospitalizations and deaths as cardiovascular, non-cardiovascular, or unknown and estimated cause-specific hazards in subsets with available diagnosis data (Item S1).

Statistical analyses were performed using R for Windows, version 4.4.2 (The R Foundation for Statistical Computing, Vienna, Austria). Statistical significance was set at the level of *P* < .05.

## RESULTS

### Patient characteristics

Among 81 475 patients contributing 431 618 landmarks (median, 5 per patient), latest and 180-day mean calcium were both 8.9 mg/dl, mean calcium slope was 0.00 mg/dl per 30 days, and mean rSD was 0.34 mg/dl (Fig. 1) (Table 1). During follow-up windows, 68 632 all-cause hospitalizations and 12 886 deaths occurred.

**Table 1:** Characteristics of the patients at the first eligible landmark, overall and by quartile of residual SD of serum calcium over the prior 180 days.

Characteristic	Missing %	Overall	Q1	Q2	Q3	Q4
		*N* = 81 475	*N* = 20 369 rSD 0.00–0.20 mg/dl	*N* = 20 369 rSD 0.20–0.29 mg/dl	*N* = 20 368 rSD 0.29–0.42 mg/dl	*N* = 20 369 rSD 0.42–2.70 mg/dl
*Demographics*						
Age at landmark, years		64.2 ± 14.6	66.8 ± 14.0	65.0 ± 14.3	63.6 ± 14.5	61.5 ± 14.9
Age at index, years		63.7 ± 14.6	66.2 ± 14.0	64.5 ± 14.3	63.1 ± 14.5	61.0 ± 14.9
Dialysis vintage at landmark, years	6.7	2.7 (1.0–5.8)	2.3 (0.9–5.1)	2.5 (0.9–5.4)	2.7 (1.0–5.7)	3.2 (1.1–6.8)
Sex						
Female		34 008 (41.8)	7964 (39.1)	8291 (40.7)	8539 (41.9)	9214 (45.2)
Male		47 445 (58.2)	12 401 (60.9)	12 073 (59.3)	11 822 (58.1)	11 149 (54.8)
Race	10.0					
Non-Black		54 040 (73.7)	14 406 (78.3)	13 849 (75.4)	13 237 (72.2)	12 548 (68.8)
Black		19 303 (26.3)	3997 (21.7)	4513 (24.6)	5096 (27.8)	5697 (31.2)
Country/region						
US/Canada		61 369 (75.3)	14 894 (73.1)	15 350 (75.4)	15 682 (77.0)	15 443 (75.8)
Japan/China		8324 (10.2)	2418 (11.9)	2266 (11.1)	1861 (9.1)	1779 (8.7)
Europe^a^		10 017 (12.3)	2657 (13.0)	2394 (11.8)	2439 (12.0)	2527 (12.4)
Other^b^		1765 (2.2)	400 (2.0)	359 (1.8)	386 (1.9)	620 (3.0)
DOPPS phase						
Phase 4 (2009–12)		12 249 (15.0)	2838 (13.9)	2982 (14.6)	3179 (15.6)	3250 (16.0)
Phase 5 (2012–15)		22 583 (27.7)	5968 (29.3)	5504 (27.0)	5410 (26.6)	5701 (28.0)
Phase 6 (2015–18)		18 447 (22.6)	4736 (23.3)	4717 (23.2)	4573 (22.5)	4421 (21.7)
Phase 7 (2018–)		28 196 (34.6)	6827 (33.5)	7166 (35.2)	7206 (35.4)	6997 (34.4)
*Comorbidities at index, n (%)*						
Coronary artery disease		18 418 (22.6)	4892 (24.0)	4738 (23.3)	4540 (22.3)	4248 (20.9)
Congestive heart failure		17 411 (21.4)	4470 (21.9)	4347 (21.3)	4374 (21.5)	4220 (20.7)
Other cardiovascular disease		15 225 (18.7)	4200 (20.6)	3810 (18.7)	3688 (18.1)	3527 (17.3)
Hypertension		62 240 (76.4)	15 513 (76.2)	15 587 (76.5)	15 600 (76.6)	15 540 (76.3)
Cerebrovascular disease		7034 (8.6)	1888 (9.3)	1765 (8.7)	1729 (8.5)	1652 (8.1)
Diabetes		44 859 (55.1)	11 447 (56.2)	11 492 (56.4)	11 348 (55.7)	10 572 (51.9)
Lung disease		6304 (7.7)	1674 (8.2)	1574 (7.7)	1579 (7.8)	1477 (7.3)
Cancer		6456 (7.9)	1791 (8.8)	1612 (7.9)	1558 (7.6)	1495 (7.3)
Neurologic disease		4992 (6.1)	1269 (6.2)	1216 (6.0)	1232 (6.0)	1275 (6.3)
Parathyroid surgery		1358 (1.7)	225 (1.1)	274 (1.3)	334 (1.6)	525 (2.6)
Prior fracture		1195 (1.5)	317 (1.6)	291 (1.4)	275 (1.4)	312 (1.5)
GI bleed		2803 (3.4)	699 (3.4)	714 (3.5)	689 (3.4)	701 (3.4)
*Serum calcium at landmark*						
Latest total Ca, mg/dl		8.9 ± 0.7	9.0 ± 0.6	9.0 ± 0.7	8.9 ± 0.7	8.9 ± 0.9
Latest albumin-corrected Ca, mg/dl	0.7	9.1 ± 0.7	9.2 ± 0.6	9.1 ± 0.6	9.1 ± 0.7	9.1 ± 0.9
Mean Ca over 180 d, mg/dl		8.9 ± 0.6	9.0 ± 0.6	8.9 ± 0.6	8.9 ± 0.6	8.9 ± 0.7
Slope of Ca 180 d, mg/dl/30 d		0.00 ± 0.14	0.00 ± 0.10	0.00 ± 0.11	0.00 ± 0.13	0.00 ± 0.19
Residual SD of Ca 180 d, mg/dl		0.34 ± 0.21	0.14 ± 0.04	0.25 ± 0.03	0.35 ± 0.04	0.62 ± 0.22
Number of Ca observations, 180 d		6 (5–6)	6 (5–6)	6 (5–6)	6 (5–6)	6 (5–6)
*Time-in-range burden (primary: 8.6–10.0 mg/dl), n (%)*						
Time-below-range—0 months		38 577 (47.3)	12 603 (61.9)	10 948 (53.7)	9102 (44.7)	5924 (29.1)
Time-below-range—1–2 months		20 810 (25.5)	3024 (14.8)	4253 (20.9)	5666 (27.8)	7867 (38.6)
Time-below-range—3–6 months		22 088 (27.1)	4742 (23.3)	5168 (25.4)	5600 (27.5)	6578 (32.3)
Time-above-range—0 months		68 441 (84.0)	19 133 (93.9)	18 382 (90.2)	17 256 (84.7)	13 670 (67.1)
Time-above-range—1–2 months		10 255 (12.6)	806 (4.0)	1480 (7.3)	2420 (11.9)	5549 (27.2)
Time-above-range—3–6 months		2779 (3.4)	430 (2.1)	507 (2.5)	692 (3.4)	1150 (5.6)
*Laboratory values at landmark*						
Albumin, g/dl	2.3	3.8 ± 0.4	3.7 ± 0.4	3.8 ± 0.4	3.8 ± 0.4	3.8 ± 0.5
Phosphate, mg/dl	0.2	5.3 ± 1.6	5.1 ± 1.5	5.2 ± 1.5	5.3 ± 1.6	5.5 ± 1.8
Hemoglobin, g/dl	1.7	10.9 ± 1.3	10.9 ± 1.2	10.9 ± 1.3	10.9 ± 1.3	10.9 ± 1.4
Creatinine, mg/dl	7.8	8.3 ± 3.0	7.8 ± 2.9	8.1 ± 3.0	8.4 ± 3.0	8.9 ± 3.1
Alkaline phosphatase, U/l	11.1	95 (71–140)	96 (73–141)	95 (71–139)	95 (71–139)	95 (70–140)
Kt/V	11.3	1.6 ± 0.3	1.6 ± 0.3	1.6 ± 0.3	1.6 ± 0.3	1.6 ± 0.3
BMI, kg/m²	4.0	27.8 ± 7.1	27.7 ± 7.0	27.9 ± 7.1	27.9 ± 7.1	27.6 ± 7.1
BUN, mg/dl	3.2	57.0 ± 18.5	56.3 ± 17.8	56.6 ± 18.0	57.2 ± 18.8	57.8 ± 19.5
PTH (90-d window), pg/ml	4.3	296 (168–496)	280 (168–447)	293 (173–475)	301 (170–506)	318 (159–573)
*PTH category (90-d window), n (%)*	4.3					
<150 pg/ml		16 572 (21.2)	4072 (20.9)	3860 (19.8)	4048 (20.8)	4592 (23.5)
150–299 pg/ml		22 900 (29.4)	6462 (33.2)	6114 (31.3)	5655 (29.0)	4669 (23.9)
300–599 pg/ml		24 680 (31.6)	6291 (32.3)	6489 (33.3)	6170 (31.7)	5730 (29.3)
≥600 pg/ml		13 847 (17.8)	2647 (13.6)	3045 (15.6)	3613 (18.5)	4542 (23.3)
*Dialysis prescription & mineral-metabolism medications*						
Dialysate Ca—Standard (2.5 mEq/l)	8.6	49 527 (66.5)	12 556 (67.9)	12 623 (68.0)	12 601 (67.3)	11 747 (62.8)
Dialysate Ca—Low (≤2.25)	8.6	8281 (11.1)	1893 (10.2)	2045 (11.0)	2122 (11.3)	2221 (11.9)
Dialysate Ca—Standard-high (2.75)	8.6	3470 (4.7)	858 (4.6)	870 (4.7)	846 (4.5)	896 (4.8)
Dialysate Ca—High (≥3.0)	8.6	13 200 (17.7)	3186 (17.2)	3026 (16.3)	3158 (16.9)	3830 (20.5)
Phosphate binder—None	0.6	18 145 (22.4)	5219 (25.8)	4787 (23.7)	4333 (21.4)	3806 (18.8)
Phosphate binder—Ca-based only	0.6	23 349 (28.8)	5433 (26.8)	5714 (28.2)	6033 (29.8)	6169 (30.5)
Phosphate binder—Non-Ca only	0.6	26 414 (32.6)	6740 (33.3)	6639 (32.8)	6643 (32.8)	6392 (31.6)
Phosphate binder—Both Ca & non-Ca	0.6	13 050 (16.1)	2856 (14.1)	3096 (15.3)	3227 (15.9)	3871 (19.1)
ESA, Yes	0.3	71 459 (87.9)	17 773 (87.4)	17 925 (88.2)	17 860 (87.9)	17 901 (88.1)
Active vitamin D receptor analogue, Yes	0.1	55 691 (68.4)	13 370 (65.7)	13 862 (68.1)	14 166 (69.6)	14 293 (70.3)
Calcimimetic, Yes	0.7	17 048 (21.1)	2928 (14.5)	3638 (18.0)	4526 (22.4)	5956 (29.5)

Values are mean ± SD, median (IQR), or n (%) as appropriate. Each patient contributes one row—the earliest landmark satisfying the inclusion criteria. Continuous missingness ≥0.1% is shown in the 'Missing %' column.

Stratification variable: rSD_Ca_180, the residual standard deviation of month-level serum total calcium over the 180 days preceding the landmark [= √(RSS/(*n* − 2)] from the within-window linear regression of Ca on time).

Abbreviations: TBR, time below range (<8.6 mg/dl); TIR, time in range (8.6–10.0 mg/dl); TAR, time above range (>10.0 mg/dl); PTH, intact parathyroid hormone; ESA, erythropoiesis-stimulating agent; VDRA, vitamin D receptor analogue; BMI, body mass index; BUN, blood urea nitrogen.

^a^Europe: Belgium, France, Germany, Italy, Spain, Sweden and UK

^b^Others: Australia-New Zealand, GCC, Russia and Turkey

### Primary outcome—90-day all-cause hospitalization

Latest total calcium was associated with a lower hazard in the minimally adjusted model (HR 0.89, *P* < .001), but not after full adjustment (HR 1.00, *P* = .964) (Table 2).

**Table 2:** Association of serum calcium metrics with hospitalization risk.Model specifications—what is adjusted in each model^a^

Characteristic	Model 1^f^ *Minimal*	Model 1-full *Latest Ca, full adj*.	Model 2^g^ *MAIN • Ca dynamics*	Model 3^h^ *TBR/TAR burden*	Model 4 *+ PTH subset*	Model 5a *Corrected Ca*
Primary exposure	Latest total Ca	Latest total Ca	Mean + Slope + rSD *(180-d total Ca)*	TBR + TAR burden *(months out of range)*	Model 2 exposures *+ PTH (90-d cat.)*	Mean + Slope + rSD *(180-d corrected Ca)*
Intervals (1–30, 31–60, 61–90 d) + offset	✓	✓	✓	✓	✓	✓
Case-mix: age, sex, race, vintage, country, phase, 12 comorbidities^b^	✓	✓	✓	✓	✓	✓
Labs (Alb, P, Hgb, Cr, ALP, Kt/V, BMI, BUN)^c^	–	✓	✓	✓	✓	✓ (excl. Alb)
Medications (binder, dialysate Ca, ESA, VDRA, calcimimetic)^d^	–	✓	✓	✓	✓	✓
Prior hospitalization (admits 31–180 d + any in last 30 d)^e^	–	✓	✓	✓	✓	✓
PTH 90-d category (<150/150–299/300–599/≥600 pg/ml)	–	–	–	–	✓	–
Facility random intercept	✓	✓	✓	✓	✓	✓
Analytic subset^i^	Complete-case	Complete-case	Complete-case	Complete-case	Complete-case, *PTH available*	Complete-case, *same-day Ca + Alb*
*N* (landmark × interval rows)	1 007 263	639 232	639 232	639 232	603 659	631 357
Events (hospitalizations)	61 685	39 372	39 372	39 372	37 251	38 797
Facilities	1543	1213	1213	1213	1199	1198
Adjusted hazard ratios by model						
Exposure (unit of change)	Model 1	Model 1-full	Model 2 (MAIN)	Model 3	Model 4	Model 5a
	*HR (95 % CI)/P*	*HR (95 % CI)/P*	*HR (95 % CI)/P*	*HR (95 % CI)/P*	*HR (95 % CI)/P*	*HR (95 % CI)/P*
*Latest total Ca (current-value exposure)*						
Latest total Ca (per +1 mg/dl)	0.89 (0.88–0.90) *P< .001*	1.00 (0.98–1.02) *P= .964*	–	–	–	–
*Ca dynamics—180-day total Ca (Models 2 and 4)*						
Mean Ca over 180 d (per +1 mg/dl)	—	–	0.97 (0.95–0.98) *P< .001*	–	0.96 (0.94–0.98) *P< .001*	–
Slope of Ca 180 d (per +1 mg/dl per 30 d)	—	–	1.10 (1.03–1.19) *P= .009*	–	1.12 (1.04–1.22) *P= .003*	–
Residual SD of Ca 180 d (per +1 mg/dl)—instability	—	–	1.15 (1.10–1.21) *P< .001*	–	1.15 (1.09–1.21) *P< .001*	–
*Ca dynamics—180-day corrected Ca (Model 5a, Alb excluded from adjustment)*						
Mean corrected Ca 180 d (per +1 mg/dl)	–	–	–	–	–	1.01 (0.99–1.03) *P= .512*
Slope of corrected Ca 180 d	–	–	–	–	–	1.08 (1.00–1.16) *P= .048*
Residual SD of corrected Ca 180 d—instability	–	–	–	–	–	1.14 (1.09–1.20) *P< .001*
*Time-in-range burden—months out of range in prior 180 d (Model 3)*						
TBR burden 1–2 months (vs. 0)	–	–	–	1.06 (1.03–1.09) *P< .001*	–	–
TBR burden 3–6 months (vs. 0)	–	–	–	1.07 (1.04–1.10) *P< .001*	–	–
TAR burden 1–2 months (vs. 0)	–	–	–	1.04 (1.01–1.07) *P= .013*	–	–
TAR burden 3–6 months (vs. 0)	–	–	–	1.02 (0.96–1.08) *P= .537*	–	–

^a^Bold HRs indicate the 95% CI excludes 1.0. Each HR is the multiplicative change in hazard per 30-day interval.

^b^Case-mix covariates (all models): age, dialysis vintage, sex, race, country/region, study phase, and comorbidities (coronary artery disease, congestive heart failure, other cardiovascular disease, hypertension, cerebrovascular disease, diabetes, lung disease, cancer, neurologic disease, prior parathyroid surgery, fracture, GI bleed).

^c^Time-varying labs (Models 1-full, 2, 3, 4, 5a): Alb, P, Hgb, Cr (most recent value within 45 d); ALP, Kt/V, BMI, BUN (most recent value within 120 d). In Model 5a, Alb is excluded from the adjustment set because it is used to compute corrected Ca [Payne formula: corrected Ca = total Ca + 0.8 × (4.0 − Alb)].

^d^Time-varying medications (Models 1-full, 2, 3, 4, 5a): phosphate-binder category (none/Ca-only/non-Ca-only/both), dialysate Ca bath (≤2.25, 2.5, 2.75, ≥3.0 mEq/l), and ESA, VDRA, calcimimetic prescription status.

^e^Prior hospitalization (Models 1-full, 2, 3, 4, 5a): mutually exclusive windows—count of admissions 31–180 days before landmark, plus a binary indicator for any admission or hospital-day in the last 30 days. (A days-based alternative is reported as m2_days in sensitivity analyses.)

^f^Model 1 is minimally adjusted (latest Ca + case-mix + facility random intercept only); comparing Model 1 with Model 1-full isolates the confounding contribution of time-varying covariates (especially Alb) to the latest-Ca risk estimate.

^g^Model 2 is the primary model of this study. Models 3–5a are planned secondary/sensitivity analyses. Model 2 restricted to the PTH-available subset and Model 4 are shown in Supplementary Table S1; Model 5b, which reintroduced albumin into the corrected-calcium model, is shown in Supplementary Table S2.

^h^TBR (time-below-range) = monthly representative Ca <8.6 mg/dl; TIR = 8.6–10.0 mg/dl; TAR = > 10.0 mg/dl. Burden is counted as the number of months (out of 4–6 observed months) in each zone during the prior 180 days.

^i^ *N* and events in Panel A refer to person-period observations and events included in each fitted model after model-specific complete-case exclusion.

Abbreviations: Ca, calcium; cCa, Payne-corrected calcium; Alb, albumin; P, phosphorus; Hgb, hemoglobin; Cr, creatinine; ALP, alkaline phosphatase; Kt/V, single-pool dialysis adequacy; BMI, body mass index; BUN, blood urea nitrogen; ESA, erythropoiesis-stimulating agent; VDRA, vitamin-D receptor activator; PTH, intact parathyroid hormone; rSD, residual standard deviation after de-trending; TBR/TIR/TAR, time below/in/above range.

In the primary Model 2, lower mean calcium, a more positive calcium slope, and greater rSD over 180 days were associated with an increased hospitalization risk (mean: HR 0.97, *P* < .001; slope: HR 1.10, *P* = .009; rSD: HR 1.15, *P* < .001, respectively). These associations were similar when Model 2 was refit on the PTH-available subset (Model 2_same: mean Ca HR 0.96, slope HR 1.10, rSD HR 1.15), and again when PTH was further added to model2 (Model 4: mean Ca HR 0.96, slope HR 1.12, rSD HR 1.15) (Table 2 and Supplementary Table S1). When calcium exposure was considered as the cumulative number of months out of range over the prior 180 days, TBR burden was associated with a graded increase in 30-day hospitalization risk (1–2 months HR 1.06, *P* < .001; 3–6 months HR 1.07, *P* < .001). TAR in the 1–2 month stratum was significantly associated with increased hospitalization risk (HR 1.04, *P* = .013), whereas TAR in the 3–6 month stratum was not (HR 1.02, *P* = .537). In corrected-calcium sensitivity analyses, mean corrected calcium was not associated with hospitalization when albumin was omitted (Model 5a: HR, 1.006; 95% CI, 0.987–1.026; *P* = .512), whereas corrected-calcium slope and rSD were positively associated. In exploratory Model 5b, in which albumin was reintroduced, mean corrected calcium showed a modest inverse association (HR, 0.973; 95% CI, 0.955–0.992; *P* = .006), rSD remained positively associated (HR, 1.139; 95% CI, 1.084–1.197; *P* < .001), whereas slope was no longer statistically significant (HR, 1.040; 95% CI, 0.964–1.122; *P* = .306) (Supplementary Table S2).

Restricted cubic spline analyses for the hospitalization outcome showed the association for latest calcium was approximately U-shaped, whereas the association for mean calcium was mainly by lower calcium levels. Greater rSD was monotonically associated with increased hospitalization risk (Fig. 3).

**Figure 3:**
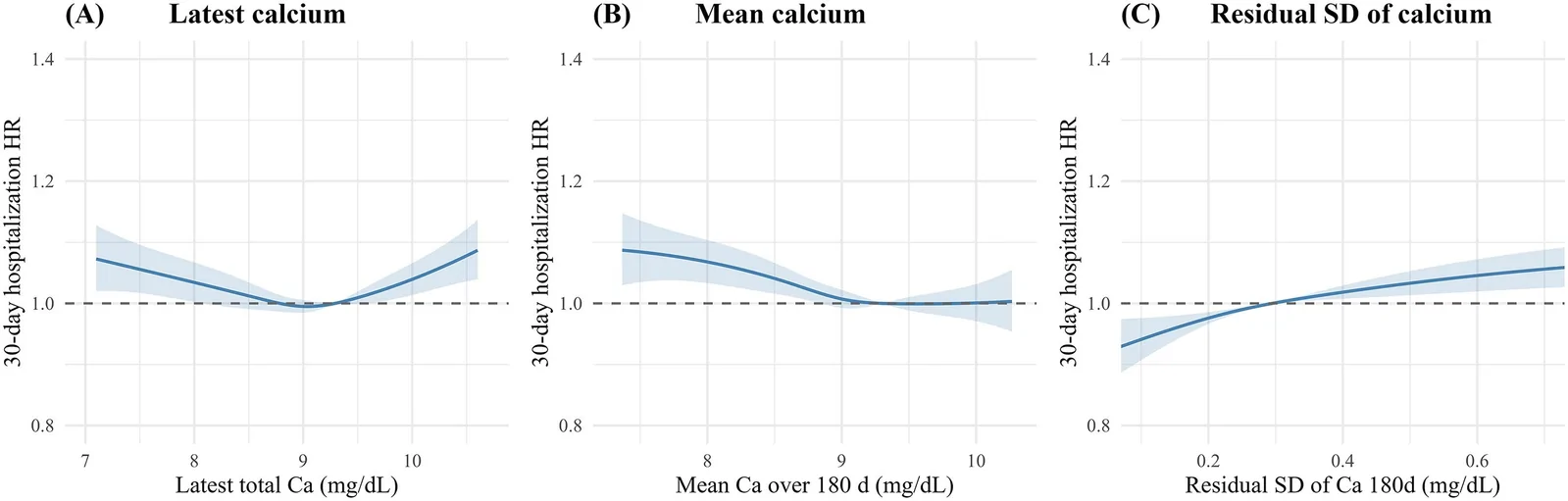
Adjusted hazard ratios for 90-day all-cause hospitalization across (A) the latest total calcium, (B) the mean total calcium over the prior 180 days, and (C) the instability of total calcium over the prior 180 days. Each exposure was fitted as a natural cubic spline over the 1st–99th percentile of its values. Solid lines are point estimates and shaded bands are 95% confidence intervals. The reference is 9.3 mg/dl for calcium concentration (panels A and B; the midpoint of the DOPPS optimal range, 8.6–10.0 mg/dl) and the median (0.30 mg/dl) for residual SD (C). Values are 30-day hazard ratios from a discrete-time (complementary log–log) survival model with a facility-level random intercept, using a quarterly dynamic landmark design with the 90-day follow-up split into 30-day intervals. Models were adjusted for case-mix and time-updated laboratory, medication, and prior-hospitalization covariates (full list in Table 2). SD, standard deviation; DOPPS, Dialysis Outcomes and Practice Patterns Study.

### Secondary outcome—90-day all-cause death

Model 1 (minimally adjusted) showed latest total calcium was significantly associated with decreased all-cause death risk (HR 0.87, *P* < .001), but, after full adjustment, the direction was reversed (HR 1.16, *P* < .001) (Table 3). Higher mean Ca, a positive slope, and greater rSD were associated with an increased all-cause mortality risk (mean, HR 1.156, *P* < .001; slope, HR 1.384, *P* < .001; rSD, HR 1.373, *P* < .001, respectively). Similar to the hospitalization analysis, these three associations were unchanged when Model 2 was refit on the PTH-available subset (Model 2_same: mean Ca HR 1.160, slope HR 1.367, rSD HR 1.366), and again when PTH was further added to model2 (Model 4: mean Ca HR 1.155, slope HR 1.304, rSD HR 1.346) (Supplementary Table S3). In time-range burden analyses, TAR burden was associated with a graded, dose-dependent increase in all-cause mortality (1–2 months HR 1.21, *P* < .001; 3–6 months HR 1.37, *P* < .001). In contrast, TBR burden was not associated with mortality at either stratum (1–2 months HR 1.04, *P* = .172; 3–6 months HR 0.94, *P* = .066).

**Table 3:** Association of serum calcium metrics with all-cause mortality risk.Model specifications—what is adjusted in each model^a^

Characteristic	Model 1^f^ *Minimal*	Model 1-full *Latest Ca, full adj*.	Model 2^g^ *MAIN • Ca dynamics*	Model 3^h^ *TBR/TAR burden*	Model 4 *+ PTH subset*	Model 5a *Corrected Ca*
Primary exposure	Latest total Ca	Latest total Ca	Mean + Slope + rSD *(180-d total Ca)*	TBR + TAR burden *(months out of range)*	Model 2 exposures *+ PTH (90-d cat.)*	Mean + Slope + rSD *(180-d corrected Ca)*
Intervals (1–30, 31–60, 61–90 d) + offset	✓	✓	✓	✓	✓	✓
Case-mix: age, sex, race, vintage, country, phase, 12 comorbidities^b^	✓	✓	✓	✓	✓	✓
Labs (Alb, P, Hgb, Cr, ALP, Kt/V, BMI, BUN)^c^	–	✓	✓	✓	✓	✓ (excl. Alb)
Medications (binder, dialysate Ca, ESA, VDRA, calcimimetic)^d^	–	✓	✓	✓	✓	✓
Prior hospitalization (admits 31–180 d + any in last 30 d)^e^	–	✓	✓	✓	✓	✓
PTH 90-d category (<150/150–299/300–599/≥600 pg/ml)	–	–	–	–	✓	–
Facility random intercept	✓	✓	✓	✓	✓	✓
Analytic subset^i^	Complete-case	Complete-case	Complete-case	Complete-case	Complete-case, *PTH available*	Complete-case, *same-day Ca + Alb*
*N* (landmark × interval rows)	1 091 848	690 451	690 451	690 451	652 086	681 789
Events (all-cause death)	11 303	6790	6790	6790	6452	6708
Facilities	1543	1213	1213	1213	1199	1198
Adjusted hazard ratios by model						
Exposure (unit of change)	Model 1	Model 1-full	Model 2 (MAIN)	Model 3	Model 4	Model 5a
	*HR (95 % CI)/p*	*HR (95 % CI)/P*	*HR (95 % CI)/P*	*HR (95 % CI)/P*	*HR (95 % CI)/P*	*HR (95 % CI)/P*
*Latest total Ca (current-value exposure)*						
Latest total Ca (per +1 mg/dl)	0.866 (0.843–0.890) *P< .001*	1.158 (1.116–1.201) *P< .001*	–	–	–	–
*Ca dynamics—180-day total Ca (Models 2 and 4)*						
Mean Ca over 180 d (per +1 mg/dl)	–	–	1.156 (1.105–1.209) *P< .001*	–	1.155 (1.102–1.211) *P< .001*	–
Slope of Ca 180 d (per +1 mg/dl per 30 d)	–	–	1.384 (1.159–1.652) *P< .001*	–	1.304 (1.085–1.568) *P= .005*	–
Residual SD of Ca 180 d (per +1 mg/dl)—instability	–	–	1.373 (1.225–1.538) *P< .001*	–	1.346 (1.196–1.514) *P< .001*	–
*Ca dynamics—180-day corrected Ca (Model 5a, Alb excluded from adjustment)*						
Mean corrected Ca 180 d (per +1 mg/dl)	–	–	–	–	–	1.290 (1.232–1.350) *P< .001*
Slope of corrected Ca 180 d	–	–	–	–	–	1.740 (1.451–2.087) *P< .001*
Residual SD of corrected Ca 180 d—instability	–	–	–	–	–	1.311 (1.166–1.474) *P< .001*
*Time-in-range burden—months out of range in prior 180 d (Model 3)*						
TBR burden 1–2 months (vs. 0)	–	–	–	1.043 (0.982–1.109) *P= .172*	–	–
TBR burden 3–6 months (vs. 0)	–	–	–	0.940 (0.879–1.004) *P= .066*	–	–
TAR burden 1–2 months (vs. 0)	–	–	–	1.213 (1.128–1.305) *P< .001*	–	–
TAR burden 3–6 months (vs. 0)	–	–	–	1.374 (1.204–1.568) *P< .001*	–	–

^a^Bold HRs indicate the 95% CI excludes 1.0. Each HR is the multiplicative change in hazard per 30-day interval.

^b^Case-mix covariates (all models): age, dialysis vintage, sex, race, country/region, study phase, and comorbidities (coronary artery disease, congestive heart failure, other cardiovascular disease, hypertension, cerebrovascular disease, diabetes, lung disease, cancer, neurologic disease, prior parathyroid surgery, fracture, GI bleed).

^c^Time-varying labs (Models 1-full, 2, 3, 4, 5a): Alb, P, Hgb, Cr (most recent value within 45 d); ALP, Kt/V, BMI, BUN (most recent value within 120 d). In Model 5a, Alb is excluded from the adjustment set because it is used to compute corrected Ca [Payne formula: corrected Ca = total Ca + 0.8 × (4.0 − Alb)].

^d^Time-varying medications (Models 1-full, 2, 3, 4, 5a): phosphate-binder category (none/Ca-only/non-Ca-only/both), dialysate Ca bath (≤2.25, 2.5, 2.75, ≥3.0 mEq/l), and ESA, VDRA, calcimimetic prescription status.

^e^Prior hospitalization (Models 1-full, 2, 3, 4, 5a): mutually exclusive windows—count of admissions 31–180 days before landmark, plus a binary indicator for any admission or hospital-day in the last 30 days. (A days-based alternative is reported as m2_days in sensitivity analyses.)

^f^Model 1 is minimally adjusted (latest Ca + case-mix + facility random intercept only); comparing Model 1 with Model 1-full isolates the confounding contribution of time-varying covariates (especially Alb) to the latest-Ca risk estimate.

^g^Model 2 is the primary model of this study. Models 3–5a are planned secondary/sensitivity analyses. Model 2 restricted to the PTH-available subset and Model 4 are shown in Supplementary Table S3; Model 5b, which reintroduced albumin into the corrected-calcium model, is shown in Supplementary Table S4.

^h^TBR (time-below-range) = monthly representative Ca <8.6 mg/dl; TIR = 8.6–10.0 mg/dl; TAR ≥ 10.0 mg/dl. Burden is counted as the number of months (out of 4–6 observed months) in each zone during the prior 180 days.

^i^ *N* and events in Panel A refer to person-period observations and events included in each fitted model after model-specific complete-case exclusion.

Abbreviations: Ca, calcium; cCa, Payne-corrected calcium; Alb, albumin; P, phosphorus; Hgb, hemoglobin; Cr, creatinine; ALP, alkaline phosphatase; Kt/V, single-pool dialysis adequacy; BMI, body mass index; BUN, blood urea nitrogen; ESA, erythropoiesis-stimulating agent; VDRA, vitamin-D receptor activator; PTH, intact parathyroid hormone; rSD, residual standard deviation after de-trending; TBR/TIR/TAR, time below/in/above range.

In corrected-calcium sensitivity analyses, higher mean corrected calcium, a more positive corrected-calcium slope, and greater corrected-calcium rSD remained associated with an increased mortality risk even after albumin was reintroduced into the model (mean corrected calcium: HR, 1.15; 95% CI, 1.10–1.20; slope: HR, 1.52; 95% CI, 1.27–1.82; rSD: HR, 1.27; 95% CI, 1.13–1.43) (Supplementary Table S4).

In restricted cubic spline analyses for the all-cause mortality outcome, both latest and mean calcium were associated with higher all-cause mortality risk. Greater calcium variability was associated with higher all-cause mortality risk (Fig. 4).

**Figure 4:**
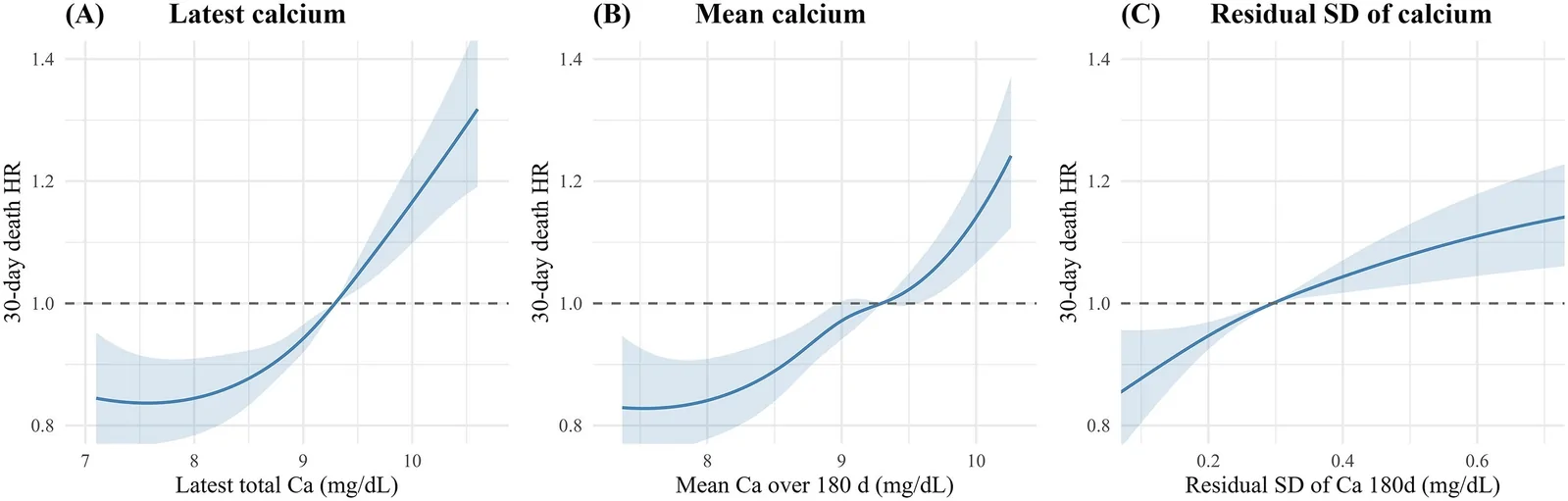
Adjusted hazard ratios for 90-day all-cause mortality across (A) the latest total calcium, (B) the mean total calcium over the prior 180 days, and (C) the instability of total calcium over the prior 180 days. Each exposure was fitted as a natural cubic spline over the 1st–99th percentile of its values. Solid lines are point estimates and shaded bands are 95% confidence intervals. The reference is 9.3 mg/dl for calcium concentration (panels A and B; the midpoint of the DOPPS optimal range, 8.6–10.0 mg/dl) and the median (0.30 mg/dl) for residual SD (C). Values are 30-day hazard ratios from a discrete-time (complementary log–log) survival model with a facility-level random intercept, using a quarterly dynamic landmark design with the 90-day follow-up split into 30-day intervals. Estimates are 30-day hazard ratios for all-cause death within 90 days of each landmark. Models were adjusted for case-mix and time-updated laboratory, medication, and prior-hospitalization covariates (full list in Table 3). SD, standard deviation; DOPPS, Dialysis Outcomes and Practice Patterns Study.

### Predictive performance of calcium dynamics versus the latest value

Cross-validated 90-day discrimination was almost identical for the latest-calcium and the dynamics models (hospitalization, AUC 0.696 vs 0.696; mortality, 0.785 vs 0.785). Entering the dynamics metrics in addition to the latest value increased the AUC by 0.0003 (95% CI, 0.0001–0.0006) for hospitalization and by 0.0007 (0.0003–0.0011) for mortality, with no improvement in the Brier score (Table S5). In these augmented models the rSD coefficient was essentially unchanged relative to Model 2 (mortality, β 0.320 vs 0.317; hospitalization, β 0.142 vs 0.140), whereas the mean and slope coefficients were markedly attenuated, consistent with the correlation of the latest value with the 180-day mean (*r* = 0.81) but not with rSD (*r* = −0.02).

### Cause-specific hospitalization and mortality

In the subset with linked diagnosis data (13 010 patients, 58 660 landmarks), 8854 first hospitalizations (1111 cardiovascular, 7072 non-cardiovascular, 671 unknown cause) and 1043 deaths (409 cardiovascular, 531 non-cardiovascular, 103 unknown cause) occurred within 90 days. For hospitalization, higher calcium was associated specifically with cardiovascular hospitalizations (latest calcium HR 1.11, 95% CI 1.02–1.21, *P* = .014; 180-day mean 1.11, 1.00–1.23, *P* = .049), whereas the corresponding estimates for non-cardiovascular hospitalizations were null. For mortality, in contrast, the excess risk associated with higher calcium was of similar magnitude for cardiovascular and non-cardiovascular death (latest calcium 1.25, 1.09–1.43 vs 1.19, 1.05–1.34; 180-day mean 1.28, 1.09–1.50 vs 1.22, 1.06–1.41; all *P* ≤ .01). Greater rSD was associated with death outcome irrespective of cause. Prolonged TAR was associated with non-cardiovascular death and showed a similar but less precise positive association with cardiovascular death (Table S6).

## DISCUSSION

In this study, recent calcium dynamics were associated with subsequent short-term outcomes, but the direction and robustness differed between hospitalization and all-cause death. A higher latest total calcium was associated with all-cause death but not hospitalization. Lower mean calcium was associated with hospitalization, whereas higher mean calcium was associated with death. In contrast, a positive slope and greater rSD were consistently associated with both hospitalization and mortality risk outcomes. Time-in-range analyses supported this outcome-specific pattern. Greater TBR showed a graded increase in hospitalization risk, whereas greater TAR was strongly and progressively associated with mortality.

For hospitalization, the adjusted spline curve for latest calcium (Fig. 3) showed a shallow U-shape, suggesting modestly higher risk at both extremes. This may explain the null linear association for the latest value (model 1-full), as the contrasting trajectories at the extremes were likely neutralized when calcium was treated as a single continuous variable. This is consistent with prior studies in which mineral-metabolism risk was driven more by hyperphosphatemia and hyperparathyroidism than by calcium alone [9, 18]. However, the spline curve for 180-day mean calcium suggested that the hospitalization signal was increased mainly by lower mean calcium levels. The association between lower mean total calcium and hospitalization may reflect short-term clinical vulnerability rather than a direct causal effect of low calcium itself. Total calcium is strongly influenced by serum albumin [19, 20]. Therefore, lower total calcium may partly capture hypoalbuminemia, malnutrition-inflammation, frailty, or acute illness burden. In addition, albumin-corrected calcium is an imperfect surrogate for ionized calcium in dialysis patients, and previous studies have reported misclassification of calcium status when corrected calcium is compared with directly measured ionized calcium [21, 22]. This interpretation is supported by our corrected-calcium sensitivity analysis, in which a mean corrected calcium was not associated with hospitalization risk. The results of spline analysis for hospitalization are also consistent with this interpretation. Higher hospitalization risk was observed at the lower range of 180-day mean total calcium, whereas sustained higher mean calcium did not show an increase in hospitalization risk. Therefore, low total calcium may be better considered as a surrogate marker for underlying frailty and increased susceptibility to hospitalization, rather than a causal factor implying that therapeutic calcium loading would attenuate this risk. Exploratory cause-specific hospitalization analyses did not identify a single cause accounting for the low-calcium association with all-cause hospitalization. Higher latest and mean calcium were associated with a cardiovascular hospitalization, whereas the estimate for mean calcium and non-cardiovascular hospitalization was modestly inverse but imprecise. The opposing directions of these point estimates may partly explain the null mean-calcium association in the diagnosis-available all-cause subset, although cohort restriction may also have contributed. Greater rSD was associated with non-cardiovascular hospitalization, while the cardiovascular estimate was positive but imprecise, consistent with the possibility that calcium instability reflects broader clinical vulnerability rather than a uniquely cardiovascular pathway.

Findings regarding mortality were more consistent. Higher latest calcium, higher mean calcium, a positive slope, greater instability, and greater TAR were all associated with increased mortality, in line with previous studies linking higher calcium to death in dialysis patients [8, 23, 24]. In DOPPS, the highest risk was seen above 10.0 mg/dl [8], and in a large nationwide cohort from Japan, higher calcium, but not lower calcium, was independently associated with mortality in baseline and time-dependent models [6, 7]. In a time-averaged analysis, excess risk at the high-calcium extreme was consistent, whereas low-calcium risk depended on concurrent phosphorus and PTH [13]. Our spline analysis showed risk rising predominantly on the high-calcium side. The reversal of the latest-calcium estimate from an inverse association in the minimally adjusted model to a positive association after full adjustment suggests substantial confounding by albumin, nutritional status, and illness severity. Our low-calcium spline findings differ in direction from the earlier DOPPS report, in which broad low calcium was null after full adjustment but severe hypocalcemia (calcium or albumin-corrected calcium ≤7.5 mg/dl), particularly with preserved albumin, was associated with higher mortality. This discrepancy may reflect treatment era and estimand. Cinacalcet use was minimal in DOPPS I–III (1.6%) but calcimimetics were prescribed to 21% of our DOPPS IV–VII cohort, and our models based on time-updated albumin, calcium-lowering treatments, and prior hospitalization. Therefore, the lower spline estimates at low calcium should not be interpreted as protective or as refuting risk from severe or transient hypocalcemia. High or rising calcium was the more robust adjusted mortality signal.

Furthermore, our time-above-range analysis showed a graded association with mortality according to the cumulative number of months above 10.0 mg/dl. This dose-dependent pattern is in line with previous target-achievement studies in which a longer duration within the recommended ranges was associated with lower mortality [6, 25]. These findings suggest that sustained or higher calcium exposure may be a clinically meaningful mortality-associated CKD-MBD signal. Several mechanisms may explain why high calcium exposure was more strongly related to death than to hospitalization. Sustained or rising calcium may reflect excessive calcium loading from calcium-based phosphate binders, vitamin D receptor activators, higher dialysate calcium prescription, low bone turnover, or impaired calcium buffering capacity. These factors may contribute to vascular and valvular calcification, arterial stiffness, myocardial vulnerability, and fatal cardiovascular events, among other adverse pathways. However, exploratory cause-specific mortality analyses did not suggest that the high-calcium mortality signal was confined to cardiovascular death. Higher latest and mean calcium and greater calcium instability were positively associated with both cardiovascular and non-cardiovascular death. These findings may indicate broader adverse pathways associated with sustained high or unstable calcium, although the non-cardiovascular category was heterogeneous and the restricted sample precludes attribution to a specific mechanism.

The association between greater calcium variability and outcomes is a novel finding. Information on calcium variability and hard outcomes is sparse. In a small single-center study including 218 hemodialysis patients, variability in calcium and PTH was associated with cardiac mortality [26]. We extend this to all-cause mortality and, to our knowledge for the first time, to short-term hospitalization in a large international cohort using a dynamic landmark design. The association between greater rSD and outcomes was maintained after adjusting for mean calcium, calcium slope, time-varying laboratory values, medications, PTH, and previous hospitalization, and also in the corrected-calcium analysis. Therefore, variability is unlikely to be explained by albumin-related variation alone and may reflect clinical instability, repeated treatment changes, fluctuating nutritional-inflammatory status, or unstable CKD-MBD control not captured by a single value or the 180-day mean. When the latest value and the dynamics metrics were entered together, the rSD coefficient was essentially unchanged for both outcomes, whereas the mean and slope coefficients were attenuated. This indicates that instability, unlike the calcium level or its short-term trend, is not captured by a single measurement. This supports a broader concept that instability of mineral-metabolism parameters, beyond absolute levels, carries prognostic information.

Some limitations should be considered. First, the observational design precludes causal inference, and residual confounding may remain. Second, monthly laboratory and medication data could not fully capture within-month fluctuations or brief treatment changes. Third, ionized calcium was unavailable. Albumin-corrected calcium, used only in sensitivity analyses, may misclassify biologically active calcium. Fourth, the 90-day horizon aligned calcium dynamics with near-term risk but cannot characterize longer-term consequences of sustained calcium exposure. Cause-specific analyses were exploratory, restricted to smaller linked subsets, and limited by unknown and heterogeneous causes. The strengths of this study include the use of a large, international and prospectively collected cohort with standardized laboratory and facility data, repeated measurements that allow longitudinal characterization of calcium exposure, and a landmark design that aligns the exposure window with subsequent short-term risk.

In conclusion, recent serum calcium dynamics were associated with short-term hospitalization and mortality in maintenance hemodialysis patients. Lower mean calcium and greater TBR were associated mainly with hospitalization, whereas higher calcium exposure and greater TAR were associated mainly with mortality. Rising calcium and greater calcium instability were associated with both outcomes. These results support longitudinal assessment of serum calcium beyond a single latest value, particularly its instability, which was not captured by a single measurement, although the gain in population-level discrimination was small. Whether interventions that reduce avoidable calcium instability or sustained high-calcium exposure improve clinical outcomes requires further study.

## Supplementary Material

sfag324_Supplemental_File

## Data Availability

Restrictions apply to the availability of the data analyzed in this study to preserve patient confidentiality. Data may be shared on request to the corresponding author with permission from the DOPPS investigators.
