# Supplementary material for: Serum calcium dynamics and risk of all-cause hospitalization and mortality in hemodialysis patients: a landmark analysis from the international DOPPS
Source: Clin Kidney J. 2026 Sep 12;19(10):sfag324. doi: 10.1093/ckj/sfag324 (PMC13627834; doi:10.1093/ckj/sfag324)
Supplement: Supplementary file 1 [file sfag324_supplemental_file.docx]

Supplementary Materials

**Supplemental Table S1.** Association of serum calcium metrics with hospitalization risk in the PTH-available subset

**Supplemental Table S2.** Adjusted hazard ratios of serum calcium metrics with hospitalization risk in corrected-calcium analyses

**Supplemental Table S3**. Association of serum calcium metrics with all-cause mortality risk in the PTH-available subset

**Supplemental Table S4.** Adjusted hazard ratios of serum calcium metrics with all-cause mortality risk in corrected-calcium analyses

**Supplemental Table S5.** Cross-validated predictive performance of latest-calcium and calcium-dynamics models

**Supplemental Table S6.** Association of serum calcium metrics with cause-specific 90-day hospitalization and mortality in the subset with linked diagnosis data

**Item S1.** Supplemental Methods

**Supplemental Table S1**. Association of serum calcium metrics with hospitalization risk in the PTH-available subset

| **Exposure / parameter** | **Model 2**  ***(primary)***  639,232 rows | **Model 2_same**  *(PTH-available subset)*  603,659 rows | **Model 4**  *(+ PTH)*  603,659 rows |
| --- | --- | --- | --- |
| **Mean Ca 180 d (per +1 mg/dL)** | **0.965 (0.947–0.984)**  *p <0.001* | **0.964 (0.945–0.983)**  *p <0.001* | **0.963 (0.944–0.982)**  *p <0.001* |
| **Slope Ca 180 d (per +1 mg/dL / 30 d)** | **1.104 (1.025–1.190)**  *p = 0.009* | **1.097 (1.016–1.184)**  *p = 0.018* | **1.125 (1.041–1.215)**  *p = 0.003* |
| **Residual SD Ca 180 d (per +1 mg/dL)** | **1.152 (1.098–1.210)**  *p <0.001* | **1.147 (1.090–1.206)**  *p <0.001* | **1.146 (1.089–1.205)**  *p <0.001* |

Point estimates are hazard ratios (HRs) with 95% confidence intervals (CIs) per 30-day interval; two-sided p values are shown on the second line.

Model 2 was refit in the PTH-available subset (Model 2_same). PTH was then added to Model 2 in the same subset (Model 4).

Abbreviations: Ca, calcium; PTH, intact parathyroid hormone; SD, standard deviation.

**Supplemental Table S2**. Adjusted hazard ratios of serum calcium metrics with hospitalization risk in corrected-calcium analyses

| **Exposure / parameter** | **Model 2**  ***(primary)***  639,232 rows | **Model 5a**  *(Corrected Ca,* *Alb excluded)*  631,357 rows | **Model 5b**  *(Corrected Ca + Alb)*  629,071 rows |
| --- | --- | --- | --- |
| **Mean Ca/cCa 180 d (per +1 mg/dL)** | **0.965 (0.947–0.984)**  *p <0.001* | 1.006 (0.987–1.026)  *p = 0.512* | **0.973 (0.955–0.992)**  *p = 0.006* |
| **Slope Ca/cCa 180 d (per +1 mg/dL / 30 d)** | **1.104 (1.025–1.190)**  *p = 0.009* | **1.079 (1.001–1.163)**  *p = 0.048* | 1.040 (0.964–1.122)  *p = 0.306* |
| **Residual SD Ca/cCa 180 d (per +1 mg/dL)** | **1.152 (1.098–1.210)**  *p <0.001* | **1.142 (1.087–1.200)**  *p <0.001* | **1.139 (1.084–1.197)**  *p <0.001* |

Point estimates are hazard ratios (HRs) with 95% confidence intervals (CIs) per 30-day interval; two-sided p values are shown on the second line. Model 2 used uncorrected total calcium and represents the primary calcium-dynamics model. Models 5a and 5b used Payne-corrected calcium, calculated as total calcium + 0.8 × (4.0 − albumin). In Model 5a, albumin was excluded from the adjustment set because corrected calcium is derived from albumin. Model 5b reintroduced albumin as an exploratory double-adjustment sensitivity analysis. Rows indicate person-period observations after model-specific complete-case exclusion. Bold indicates 95% CIs excluding 1.0.

Abbreviations: Alb, albumin; Ca, calcium; cCa, Payne-corrected calcium; CI, confidence interval; HR, hazard ratio; SD, standard deviation.

**Supplemental Table S3**. Association of serum calcium metrics with all-cause mortality risk in the PTH-available subset

| **Exposure / parameter** | **Model 2**  ***(primary)***  690,451 rows / 6,790 deaths | **Model 2_same**  *(PTH-available subset)*  652,086 rows / 6,452 deaths | **Model 4**  *(+ PTH)*  652,086 rows / 6,452 deaths |
| --- | --- | --- | --- |
| **Ca dynamics** |  |  |  |
| **Mean Ca 180 d (per +1 mg/dL)** | **1.156 (1.105–1.209)**  *p <0.001* | **1.160 (1.107–1.215)**  *p <0.001* | **1.155 (1.102–1.211)**  *p <0.001* |
| **Slope Ca 180 d (per +1 mg/dL / 30 d)** | **1.384 (1.159–1.652)**  *p <0.001* | **1.367 (1.139–1.641)**  *p <0.001* | **1.304 (1.085–1.568)**  *p = 0.005* |
| **Residual SD Ca 180 d (per +1 mg/dL)** | **1.373 (1.225–1.538)**  *p <0.001* | **1.366 (1.215–1.536)**  *p <0.001* | **1.346 (1.196–1.514)**  *p <0.001* |

Point estimates are hazard ratios (HRs) with 95% confidence intervals (CIs) per 30-day interval; two-sided p values are shown on the second line.

Model 2 was refit in the PTH-available subset (Model 2_same). PTH was then added to Model 2 in the same subset (Model 4).

Abbreviations: Ca, calcium; PTH, intact parathyroid hormone; SD, standard deviation.

**Supplemental Table S4**. Adjusted hazard ratios of serum calcium metrics with all-cause mortality risk in corrected-calcium analyses

| **Exposure / parameter** | **Model 2**  ***(primary)***  690,451 rows | **Model 5a**  *(Corrected Ca,* *Alb excluded)*  681,789 rows | **Model 5b**  *(Corrected Ca + Alb)*  679,345 rows |
| --- | --- | --- | --- |
| **Mean Ca/cCa 180 d (per +1 mg/dL)** | **1.156 (1.105–1.209)**  *p <0.001* | **1.290 (1.232–1.350)**  *p <0.001* | **1.148 (1.096–1.202)** *p <0.001* |
| **Slope Ca/cCa 180 d (per +1 mg/dL / 30 d)** | **1.384 (1.159–1.652)**  *p <0.001* | **1.740 (1.451–2.087)** *p <0.001* | **1.519 (1.266–1.822)** *p <0.001* |
| **Residual SD Ca/cCa 180 d (per +1 mg/dL)** | **1.373 (1.225–1.538)**  *p <0.001* | **1.311 (1.166–1.474)** *p <0.001* | **1.273 (1.131–1.432)**  *p <0.001* |

Point estimates are hazard ratios (HRs) with 95% confidence intervals (CIs) per 30-day interval; two-sided p values are shown on the second line. Model 2 used uncorrected total calcium and represents the primary calcium-dynamics model. Models 5a and 5b used Payne-corrected calcium, calculated as total calcium + 0.8 × (4.0 − albumin). In Model 5a, albumin was excluded from the adjustment set because corrected calcium is derived from albumin. Model 5b reintroduced albumin as an exploratory double-adjustment sensitivity analysis. Rows indicate person-period observations after model-specific complete-case exclusion. Bold indicates 95% CIs excluding 1.0.

Abbreviations: Alb, albumin; Ca, calcium; cCa, Payne-corrected calcium; CI, confidence interval; HR, hazard ratio; SD, standard deviation.

**Supplemental Table S5.** Cross-validated predictive performance of latest-calcium and calcium-dynamics models

| **Outcome** | **Model** | **90-day AUC** | **ΔAUC vs latest Ca** | **Brier score** | **Brier gain** |
| --- | --- | --- | --- | --- | --- |
| **Hospitalization** | Latest Ca (Model 1-full) | 0.6960 | Reference | 0.13354 | Reference |
|  | Dynamics (Model 2) | 0.6963 | 0.00033 (0.00009 to 0.00057) | 0.13353 | 0.000008 (−0.000015 to 0.000030) |
|  | Augmented | 0.6963 | 0.00033 (0.00008 to 0.00061) | 0.13353 | 0.000012 (−0.000012 to 0.000038) |
| **Mortality** | Latest Ca (Model 1-full) | 0.7848 | Reference | 0.02724 | Reference |
|  | Dynamics (Model 2) | 0.7855 | 0.00066 (0.00025 to 0.00110) | 0.02724 | 0.000002 (−0.000010 to 0.000013) |
|  | Augmented | 0.7855 | 0.00066 (0.00031 to 0.00106) | 0.02724 | 0.000004 (−0.000006 to 0.000014) |

Values are out-of-fold estimates from patient-grouped five-fold cross-validation, averaged across folds with weights proportional to the number of landmarks; 95% confidence intervals are from 1,000 patient-cluster bootstrap resamples. The augmented model adds the 180-day mean, slope and rSD to the latest calcium value. Positive ΔAUC and positive Brier gain both denote improvement over the latest-calcium model. Analysis sets: hospitalization, 239,347 landmarks / 639,232 person-period rows / 39,372 events; mortality, 239,347 landmarks / 690,451 rows / 6,790 deaths.

**Supplemental Table S6.** Association of serum calcium metrics with cause-specific 90-day hospitalization and mortality in the subset with linked diagnosis data

| **Exposure** | **All-cause** | **Cardiovascular** | **Non-cardiovascular** |
| --- | --- | --- | --- |
| **Hospitalization** |  |  |  |
| **Events** | **8,854** | **1,111** | **7,072** |
| Model 1-full: Latest Ca | 1.025 (0.993–1.057)  p = 0.127 | **1.114 (1.022–1.214)**  p = 0.014 | 1.008 (0.973–1.043)  p = 0.672 |
| Model 2: Mean Ca 180 d | 0.995 (0.958–1.034)  p = 0.801 | **1.111 (1.001–1.233)**  p = 0.049 | 0.974 (0.933–1.016)  p = 0.225 |
| Model 2: Slope Ca 180 d | 1.084 (0.930–1.263)  p = 0.303 | 1.444 (0.942–2.215)  p = 0.092 | 1.023 (0.863–1.213)  p = 0.794 |
| Model 2: Residual SD Ca 180 d | **1.158 (1.048–1.280)**  p = 0.004 | 1.127 (0.852–1.491)  p = 0.401 | **1.190 (1.065–1.329)**  p = 0.002 |
| Model 3: TBR 1–2 months | 1.027 (0.972–1.086)  p = 0.342 | 1.004 (0.862–1.171)  p = 0.954 | 1.035 (0.972–1.102)  p = 0.281 |
| Model 3: TBR 3–6 months | 1.015 (0.959–1.075)  p = 0.603 | 0.923 (0.789–1.080)  p = 0.319 | 1.037 (0.973–1.105)  p = 0.260 |
| Model 3: TAR 1–2 months | 1.059 (0.990–1.132)  p = 0.094 | **1.197 (1.003–1.429)**  p = 0.047 | 1.042 (0.967–1.124)  p = 0.279 |
| Model 3: TAR 3–6 months | 0.995 (0.890–1.113)  p = 0.931 | 0.984 (0.718–1.348)  p = 0.920 | 0.984 (0.869–1.115)  p = 0.805 |
| **Mortality** |  |  |  |
| **Events** | **1,043** | **409** | **531** |
| Model 1-full: Latest Ca | **1.236 (1.134–1.348)**  p = < 0.001 | **1.249 (1.089–1.432)**  p = 0.001 | **1.187 (1.053–1.339)**  p = 0.005 |
| Model 2: Mean Ca 180 d | **1.274 (1.150–1.413)**  p = < 0.001 | **1.277 (1.085–1.503)**  p = 0.003 | **1.224 (1.061–1.413)**  p = 0.006 |
| Model 2: Slope Ca 180 d | 1.449 (0.948–2.215)  p = 0.087 | 1.671 (0.855–3.266)  p = 0.133 | 1.179 (0.646–2.151)  p = 0.592 |
| Model 2: Residual SD Ca 180 d | **1.708 (1.311–2.224)**  p = < 0.001 | **1.802 (1.192–2.724)**  p = 0.005 | **1.565 (1.073–2.283)**  p = 0.020 |
| Model 3: TBR 1–2 months | 1.025 (0.874–1.202)  p = 0.761 | 1.122 (0.878–1.435)  p = 0.358 | 0.915 (0.726–1.153)  p = 0.451 |
| Model 3: TBR 3–6 months | **0.822 (0.696–0.970)**  p = 0.020 | **0.748 (0.573–0.975)**  p = 0.032 | 0.883 (0.703–1.109)  p = 0.283 |
| Model 3: TAR 1–2 months | **1.276 (1.056–1.541)**  p = 0.012 | 1.184 (0.875–1.601)  p = 0.273 | 1.298 (0.993–1.695)  p = 0.056 |
| Model 3: TAR 3–6 months | **1.607 (1.227–2.105)**  p = < 0.001 | 1.537 (1.000–2.362)  p = 0.050 | **1.571 (1.073–2.299)**  p = 0.020 |

Values are hazard ratios (95% confidence intervals) per 30-day interval from discrete-time complementary log–log models with a facility random intercept, adjusted for the same covariates as the corresponding main-analysis models. Analysis set for both panels: 13,010 patients, 58,660 landmarks, 567 facilities. Continuous calcium metrics are per +1 mg/dL (slope per +1 mg/dL per 30 days); burden categories are versus 0 months. Cause was determined from the admission initiating each hospitalization spell and from the DOPPS cause-of-death file, respectively. Cause-specific hazards were estimated by censoring follow-up when an event of another cause occurred, including 671 hospitalizations (7.6%) and 103 deaths (9.9%) of unknown cause, which were treated as a separate competing event. Both panels use the three-level burden categories of the main analysis; PTH, corrected-calcium and spline analyses were not repeated. Bold indicates a 95% CI excluding 1.0.

**Item S1. Supplemental Methods**

**S1.1. Discrete-time survival model specification**

For landmark i and interval j (j = 1, 2, 3, corresponding to days 1–30, 31–60, and 61–90), the conditional probability of an event in interval j, p_ij, was modeled as

cloglog(p_ij) = α_j + x_iᵀβ + u_facility(i) + log(days_at_risk_ij / 30),

where α_j is an interval-specific intercept (categorical interval indicator with days 1–30 as the reference), x_i is the vector of fixed and time-varying covariates evaluated at landmark i, u_facility(i) is a normally distributed facility-level random intercept, and the offset log(days_at_risk_ij / 30) re-expresses the linear predictor on a 30-day hazard scale and accommodates partial follow-up within an interval. The exponentiated coefficient exp(β) was interpreted as the HR per 30-day interval, with 95% CIs from asymptotic standard errors. In the primary inferential models, the continuous calcium metrics were entered as linear terms to provide interpretable hazard ratios. In pre-specified secondary analyses, natural cubic splines were used to model potentially nonlinear associations for the latest total calcium and the 180-day mean calcium (4 degrees of freedom each) and for the 180-day residual SD of calcium (3 degrees of freedom); fitted hazard ratios were plotted across the 1st–99th percentile of each exposure, relative to a reference of 9.3 mg/dL (the midpoint of the previously reported optimal range, 8.6–10.0 mg/dL) for the calcium concentrations and to the empirical median for the residual SD.

**S1.2. Covariate**

Case-mix covariates included age at landmark, dialysis vintage at landmark, sex, race (Black vs non-Black), country/region (US/Canada, Japan/China, Europe, other), DOPPS phase, and baseline comorbidities (coronary artery disease, congestive heart failure, other cardiovascular disease, hypertension, cerebrovascular disease, diabetes, lung disease, cancer, neurologic disease, prior parathyroid surgery, prior fracture, and gastrointestinal bleeding).

Time-varying laboratory covariates were the most recent values within 45 days of the landmark for albumin, phosphate, hemoglobin, and creatinine, and within 120 days for alkaline phosphatase, single-pool Kt/V, body mass index, and blood urea nitrogen. Time-varying medication covariates included phosphate-binder category (none, calcium-based only, non-calcium-based only, or both), dialysate calcium concentration (low <=2.25, standard 2.5, standard-high 2.75, or high >=3.0 mEq/L), and current use of erythropoiesis-stimulating agents, vitamin D receptor activators, and calcimimetics. Prior hospitalization was captured using the number of admissions 31-180 days before the landmark and a binary indicator of any admission or hospital day within the preceding 30 days.

**S1.3. Missing data**

Models were fitted using complete-case analysis for variables included in each model. Multiple imputation was not performed because calcium-dynamics metrics were derived from repeated, time-updated measurements within prespecified landmark windows, and imputation would have required assumptions that could distort temporal structure and within-patient variability. Missingness at the landmark was below 5% for albumin, phosphate, hemoglobin, body mass index, phosphate-binder category, erythropoiesis-stimulating agents, vitamin D receptor activators, calcimimetics, and calcium-dynamics features, and was higher for alkaline phosphatase, single-pool Kt/V, and PTH; missingness rates are summarized in **Table1**.

**S1.4. Predictive performance analysis.**

Three models with identical covariates were fitted on the identical complete-case landmark set for each outcome: Model A, full covariates plus the latest total calcium (Model 1-full); Model B, full covariates plus the 180-day mean, slope and rSD (Model 2); and Model C, full covariates plus all four calcium metrics. Because the person-period file omits intervals after an event or censoring, a prediction grid containing all three 30-day intervals was reconstructed for every landmark, with days at risk fixed at 30 (offset zero) and the facility random intercept set to zero; predictions are therefore conditional on a facility random intercept of zero rather than marginal. With the complementary log–log link, the 90-day risk is 1 − Π(1 − p_j) = 1 − exp(−Σ exp(η_j)). Discrimination was summarized by the 90-day cumulative/dynamic time-dependent AUC and overall accuracy by the 90-day Brier score, both using inverse-probability-of-censoring weights estimated by Kaplan–Meier; death was treated as censoring in the hospitalization analysis, matching the main model, and censoring was administrative only in the mortality analysis. Internal validation used patient-grouped five-fold cross-validation in which all landmarks from a patient were assigned to the same fold; performance was computed within folds and averaged with weights proportional to the number of landmarks. Ninety-five percent confidence intervals and paired differences were obtained from 1,000 patient-cluster bootstrap resamples of the out-of-fold predictions. Because the primary quantity is the paired difference between models sharing the same covariates, the absolute calibration of these fixed-effects-only predictions is not interpreted.

**S1.5. Cause-specific analyses.**

Using the subset with linked diagnosis data (cause of hospitalization and cause of death) of 13,010 patients contributing 58,660 eligible landmarks after complete-case exclusion, we performed cause-specific analyses. Cardiovascular hospitalization and death were defined by outcome of interest caused by angina, acute myocardial infarction, cardiac arrest or sudden death, congestive heart failure, cardiomyopathy, valvular heart disease, atrial fibrillation, other arrhythmia, pericarditis or tamponade, transient ischaemic attack, ischaemic stroke, subdural haematoma, aortic aneurysm, or deep vein thrombosis. Events with no recorded cause were classified as unknown. Cause-specific hazards were estimated by censoring follow-up at the occurrence of an event of another cause, including unknown cause; because death is terminal, follow-up for the cause-specific mortality models ends at the date of a competing death by construction. Death and the end of hospitalization data collection were otherwise handled as in the corresponding primary analyses. Both cause-specific analyses used the same three-level time-in-range burden categories as the main analysis. All models retained the facility random intercept.
